# Diseases of the Lungs and Pleuræ, Including Consumption

**Published:** 1887-04

**Authors:** 


**Diseases of the Lungs and Pleuræ, Including Consumption.** By R. DOUGLAS POWELL, M. D., Lond., Fellow of the Royal College of Physicians; Physician to the Middlesex Hospital and to the Hospital for Consumption and Diseases of the Chest, at Brompton; late Assistant Physician and Lecturer on Materia Medica at the Charing Cross Hospital. Third edition, re-written and enlarged, with illustrations, including two lithographic plates; being Vol. XI. of Wood's Library for 1886. New York: William Wood & Co.

